# Prevalence of smear positive pulmonary tuberculosis and associated risk factors among prisoners in Hadiya Zone prison, Southern Ethiopia

**DOI:** 10.1186/s13104-016-2005-7

**Published:** 2016-04-02

**Authors:** Terefe G. Fuge, Samuel Y. Ayanto

**Affiliations:** Department of Medical Laboratory Sciences, Hossana College of Health Sciences, P.O. Box 159, Hossana, Ethiopia; Department of Public Health, Hossana College of Health Sciences, Hossana, Ethiopia

**Keywords:** Smear positive pulmonary tuberculosis, Prisoners

## Abstract

**Background:**

People concentrated in congregated systems such as prisons, are important but often neglected reservoirs for tuberculosis transmission, and threaten those in the outside community. The condition is more serious in Africa particularly in Sub-Saharan Africa (SSA) due to its poor living conditions and ineffective health services.

**Objectives:**

This study was conducted to determine the prevalence of smear positive pulmonary tuberculosis and associated risk factors among prisoners in Hadiya Zone prison.

**Methods:**

A cross-sectional survey was carried out from May to June 2013 in Hadiya Zone prison. Prison inmates who had history of cough for at least a week were included in the study. Three morning sputum samples were collected from suspected inmates and examined through compound light microscopy. The data obtained was analyzed using statistical software like Epidata and STATA.

**Results:**

A total of 164 prisoners were included in the survey using active screening strategy and the point prevalence of smear positive pulmonary tuberculosis (PTB) in the prison was 349.2 per 100,000 populations; about three times higher than its prevalence in the general population. Even though lack of visit from family was the only variable identified as a risk factor for PTB (P = 0.029), almost all of the PTB positive cases were rural residents, farmers, male youngsters and those who shared cell with TB patients and chronically coughing persons as well as those who stayed in a cell that contains >100 inmates.

**Conclusion:**

There is high prevalence of TB in Hadiya Zone prison with possible active transmission of TB within the prison. The study also documented a number of factors which may facilitate exposures to TB though most of them are not significantly associated. Therefore, strong cooperation between prison authorities and the national tuberculosis control programmes is urgently required to develop locally appropriate interventions to reduce transmission.

## Background

Tuberculosis (TB) remains a major global public health problem. It causes ill-health among millions of people each year and ranks as the second leading cause of death from infectious diseases worldwide, after HIV. WHO estimated that there were almost 8.7 million new cases in 2011 and 1.4 million TB deaths. Asia and Africa account for 60 and 24 % of cases globally, respectively [1]. The Sub-Saharan Africa (SSA) countries have the highest rates, with an average rate of about 300 per 100,000 populations [2].

Ethiopia ranks 7th among the 22 high tuberculosis burden countries. It is one of the top three in Africa with regard to a number of TB patients. According to WHO estimates in 2012, the incidence rate of all forms of TB in Ethiopia was 258 per 100,000 and 105 new smear positive cases/100,000; in the same year, the prevalence of TB infections was 237/100,000 and the mortality rate due to TB was 18/100,000 [3, 4].

The situation is more severe in prisons. Most studies conducted on TB in prisons showed high prevalence rate and it usually exceeds prevalence rates in the specific country substantially. WHO estimated 10–100 fold higher prevalence of TB in prisons than the general population [5]. In SSA, it is estimated to be 6–30 times higher than that in the general population [3]. A study in Zambia indicated, about tenfold higher TB prevalence in prisons than in the general population [6]. In Cameroon, it was 35 times higher than in the general population [7]. The point prevalence of pulmonary TB in three major prison settings of eastern Ethiopia was 1913/100,000 which is seven times higher than in the general population [8]. In the study in North Gondar Zone Prison, the point prevalence of smear positive TB was 1482.3 per 100,000 populations which was 9.1 times higher than it was in the general population [9].

A number of factors provoke tuberculosis in prisons. The main risk factors identified by most studies include low education, homelessness, low income, excess alcohol use, limited access to health care, overcrowding, length of imprisonment, re-imprisonment and high HIV prevalence [10–12]. The situation is the worst in SSA where most of the prisoners come from underprivileged groups in the general population and more risky prisons environment. TB in prisons encompasses not only TB in prisons, but also TB in prison staff who ultimately interact directly with their families and community when they are back from work. Therefore prison health is a critical part of public health as health problems within and outside prisons are interrelated [13].

The lack of specific and integrated interventions in prison can make the settings to be amplification sites, including multi drug resistant TB (MDR-TB), since a late case detection, inadequate treatment of infectious cases, release and recidivism without screening protocol, overcrowding, and poor ventilation are likely apparent characteristics of Ethiopian prisons. However, information about epidemiology of TB in prison is very limited. Thus, this epidemiological study determined prevalence and associated risk factors for pulmonary tuberculosis in Hadiya Zone prison and hence it facilitates decision making about how to screen TB, prevent further spread and provide appropriate prevention and control measures. It has substantial contribution for developing and implementing TB control program in prison. Moreover, it gives an opportunity to detect and manage those undiagnosed TB cases, and reduces potential sources of transmission for the prison and general population.

## Methods

### Study area

The study was conducted in Hossana town in Southern Ethiopia which is situated 232 km Southwest of Addis Ababa with an average elevation of 2276 m above sea level. The town has a total area of 23 km^2^ and lies between 7^o^33′N latitude and 37 ^o^51′06.67″E longitude. Its total population is estimated to be 89,300 in 2011 [14]. Hadiya zone prison is located in the town and accommodates hundreds of inmates every year. According to the information obtained from the Hadiya Zone prison authority the prison has 859 inmates currently. The prison has a clinic for the inmates and the prison staff. It is meant for the diagnosis and treatment of infectious and non-infectious diseases according to National Tuberculosis Control Program (NTCP) protocol [1]. The prison clinic is being regularly supervised and supported by Zonal Health Department. It also provides service for the diagnosis and treatment of TB and HIV.

### Study design

A cross sectional study was conducted from May to June 2013 in Hadiya Zone prison to determine the prevalence of smear positive pulmonary TB and its associated risk factors.

### Sampling method and sampling procedure

A mass screening strategy was used to identify pulmonary tuberculosis (PTB) suspects. This provided an equal chance of selecting eligible individuals, and reduced a chance of losing PTB suspects. First, a complete registration was made for all prisoners who had just a cough. The registration was done by trained nurses. It was conducted through visiting cell to cell. Secondly, all those who had cough were interviewed whether or not they fulfill the inclusion criteria. Prisoners who were mentally fit, willing to participate, above or equal to 15 years old and has ≥1 week duration of cough were included in the study. In addition, PTB patients, who were taking anti-TB treatment, were also included in the study.

### Collection of inmates’ information

A structured questionnaire was used for collecting data from inmates having TB symptoms. The questionnaire had four parts; socio-demographic and behavioural information, prison history and condition, and medical history. It had mainly closed type of questions that are commonly used in cross-sectional studies [15]. The questionnaire was prepared in English and Amharic languages, and was translated to Hadiyisa (local language). After completion of data it was translated back to English. So, the participants were interviewed with their mother language. As a result, the knowledge of the local language was considered for selecting data collectors. Two days training was given on basic techniques of interview and data collection.

### Collection and handling of sputum specimen

Three early morning sputum specimens were collected on three consecutive days using coded and clean plastic containers by laboratory personnel according to WHO guidelines on sputum collection procedure [13]. The collected specimen was pooled in one container and daily transported using ice box to the laboratory for smear microscopy.

### Direct smear microscopy of the sputum

The common staining technique, (Ziehl–Neelsen) procedure for direct smear microscopy was used according to NTCP protocol [1]. A positive result indicates the presence of AFB in the specimen and the individual tested is considered to have smear positive PTB. A negative result in this method indicates that no acid fast bacilli are seen in 100 fields and the individual tested is considered to have no smear positive PTB. All sputum specimens were stained and examined in the microscope (100 fields) by trained laboratory technicians. It was conducted at a laboratory of Hossana College of Health Sciences.

### Ethical considerations

Consent was sought from Hdiya Zone prison authority and written informed consent was obtained from the study subjects. The study also obtained ethical clearance from the research committee of Hossana College of Health Sciences. Smear positive patients for TB were put on treatment by the prison staff following the national standard clinical management protocol.

### Data analysis

The data obtained from the study was computerized using Epidata version 3.1 data entry format and exported to statistical software, STATA version 11 for analysis. Means and standard deviations were calculated for continuous variables while odds ratio (OR) was calculated to check statistical association between the dependent and independent variables using the binary logistic regression. All statistical tests and generalizations were done by assuming 95 % confidence interval and 5 % level of significance.

## Results

### Socio-demographic and behavioural factors

**Fig. 1:**
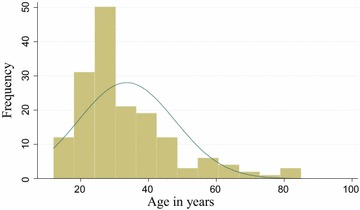
Age distribution of the inmates in Hadiya Zone prison from May to June 2013

There were a total of 164 inmates with cough of 1 week duration and more and all of them were included in the study. Of these, 161 (98.2 %) were males and 94 (57.3 %) were inmates between 20–44 years of age with the median age of 48 (Fig. 1).

Concerning marital status before imprisonment, 106 (64.6 %) prisoners were married and 57 (34.8 %) them were single. 35 (21.3 %) of them were not able to read and write or illiterate, but 124 (75.6 %) of them have completed the primary or secondary schools. Farmers account the highest percentage of the prisoners, 97 (59.2 %), followed by private employees, 38 (23.2 %). Majority of the prisoners 118 (72 %) were rural residents and the rest 46 (28 %) were urban residents (Table 1). During the study, 90 (54.9 %) prisoners were smoking cigarette. The median duration of smoking was 9 years with more than half of the smokers were smoking for the last 2 years.

**Table 1:** Univariate analysis of the association between smear positive PTB and socio-demographic and behavioral factors among the prison population in Hadiya Zone from May to June 2013

Variable	Label	No. tested (%)	No. positive (%)	No. negative (%)	Crude OR (95 % CI)	P value
Sex	Female	3 (1.8)	0	3 (100)	–	0.811
	Male	161 (98.2)	3 (1.9)	159 (98.1)	–	
Age (in years)	12–24	43 (26.2)	1 (2.3)	42 (97.7)	1.0	0.585
	25–34	60 (36.6)	2 (3.3)	58 (96.7)	1.46 (0.33–5.88)	
	35–44	34 (20.7)	0	34 (100)	–	
	>44	27 (16.5)	0	27 (100)	–	
Marital status before imprisonment	Single	57 (34.8)	1 (1.8)	56 (98.2)	1.0	0.989
	Married	106 (64.6)	2 (1.9)	104 (98.1)	1.05 (0.278–4.33)	
Educational status	Uneducated	36 (22)	0	36 (100)	–	0.804
	Primary	107 (65.2)	3 (2.8)	104 (97.2)	–	
	Secondary	17 (10.4)	0	17 (100)	–	
	C. graduate	4 (2.4)	0	4 (100)	–	
Occupation before imprisonment	G. employee	7 (4.3)	0	7 (100)	–	0.909
	Farmer	97 (59.2)	3 (3.1)	94 (96.9)	–	
	P. employee	38 (23.2)	0	38 (100)	–	
	Student	13 (7.9)	0	13 (100)	–	
	House wife	2 (1.2)	0	2 (100)	–	
	Others	6 (3.7)	0	6 (100)	–	
	Unemployed	1 (0.6)	0	1 (100)		
Residence place	Rural	118 (72)	3 (2.5)	115 (97.5)	–	0.275
	Urban	46 (28)	0	46 (100)	–	
Cigarette smoking	No	74 (45.1)	1 (1.4)	73 (98.6)	1	0.45
	Yes	90 (54.9)	2 (2.2)	88 (97.8)	2.47 (0.61–10.072)	

The point prevalence of smear positive TB in Hadiya Zone Prison was 349.2 per 100,000 populations and all the smear positive PTB cases were male farmers who were jailed from rural areas. The proportion of smear positive PTB cases among 25–34 years of age group was higher as compared to it among 12–24 and above 34 years of age group. However, both associations were not statistically significant (P = 0.585 and OR = 1.46). Two third of smear positive PTB cases were cigarette smokers though it was not significantly associated (P = 0.45 and OR = 2.47) (Table 1).

### Prison related factors

As to the length of staying in custody, 141 (86 %) prisoners stayed for ≤2 years in the current prisons. The median duration of staying was 15 months. The majority of them, 160 (97.6 %), were imprisoned for the first time, while 4 (2.4 %) prisoners had been incarcerated more than once. In addition, 7 (4.3 %) of them had a history of imprisonment in another prison. Nevertheless, more than half of the prisoners 88 (53.7 %) were jailed longer than half a month in police stations during a pre-trial period. Seventy one (43.3 %) prisoners reported of sharing a cell with a pulmonary TB patient. The median duration of sharing was 11.4 months. Also, 148 (90.2 %) of them were sharing a cell with a chronically coughing person. The median duration of sharing was 10.9 months. Besides, 114 (69.5 %) of them were sharing eating and drinking materials, such as cups, plastic bottles, and plates.

**Fig. 2:**
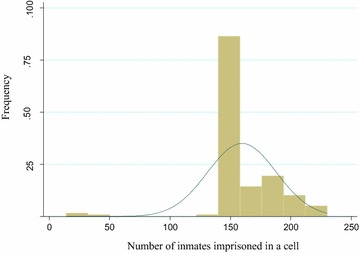
Distribution of prisoners per cell in Hadiya Zone prison from May to June 2013

Figure 2 shows density of prisoners per cell, which demonstrates a wide range of distribution, i.e. 14–230 prisoners per cell in fact the area of each cell is not the same. The mean number of prisoners per cell was 160. Eighty two (50 %) of them were in a cell that had >150 prisoners, and only three (1.8 %) of them were in a cell that had ≤50 prisoners, of course, all of them were females (Table 2).

**Table 2:** Univariate analysis of the association between smear positive PTB and prison factors among the inmates in Hadiya Zone prison from May to June 2013

Variable	Label	No. tested (%)	No. positive (%)	No. negative (%)	Crude OR (95 % CI)	P value
Length of staying	≤2 years	141 (86)	2 (1.4)	139 (98.6)	1.0	0.356
	>2 year	23 (14)	1 (4.4)	22 (95.6)	3.158 (0.426–23.434)	
Frequency of imprisonment	Once	160 (97.6)	3 (1.9)	157 (98.1)	–	0.782
	Twice	4 (2.4)	0	4 (100)	–	
Previous imprisonment in other prison	No	157 (95.7)	3 (1.9)	154 (98.1)	–	0.712
	Yes	7 (4.3)	0	7 (100)	–	
Sharing cell with TB patient	No	37 (22.6)	0	37 (100)	–	0.584
	Yes	71 (43.3)	2 (2.8)	69 (97.2)	1.595 (0.391–6.50)	
	Don’t know	56 (34.1)	1 (1.8)	55 (98.2)	1.0	
Sharing cell with chronically coughing person	No	16 (9.8)	0	16 (100)	–	0.565
	Yes	148 (90.2)	3 (2)	145 (98)	–	
Number of prisoners per cell	≤100	3 (1.8)	0	3 (100)	–	0.811
	>100	161 (98.2)	3 (1.9)	158 (98.1)	–	
Sharing food and drink materials	No	50 (30.5)	1 (2)	49 (98)	1.0	0.914
	Yes	114 (69.5)	2 (1.8)	112 (98.2)	0.875 (0.216–3.542)	
Visit from family	No	64 (39)	3 (4.7)	61 (95.3)	–	0.029**
	Yes	100 (61)	0	100 (100)	–	

** P < 0.05

All of the study population resides in a cell that had window. Almost all of them 161 (98.2 %) always open the windows during day and night times but women’s cell window was not open at all. There was a permission to be outside of a cell starting from 7:30 am till 17:00 pm every day and 146 (89 %) of the prisoners usually stayed outside of their cells and 18 (11 %) of them spent less time outside of their cells.

Visit from family was significantly associated with smear positive PTB (P = 0.029), with higher prevalence recorded among those who had no visit than those who were visited once and more per month. Smear positive PTB cases were higher also in prisoners who stayed for more than 2 years in prison and those who shared cell with chronically coughing person and TB patient but the association was not significant. Prisoners who were imprisoned in a cell having more than 100 inmates and those who were sharing food and drink materials with others had higher risk of acquiring PTB even though the association was not significant (Table 2).

### Morbidity related factors

**Fig. 3:**
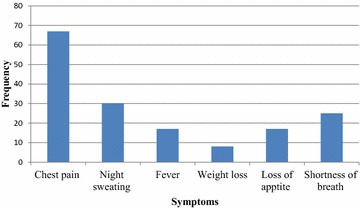
Proportion of TB symptoms among the inmates in Hadiya Zone Prison from May to June 2013

Among the complaints reported by both PTB suspects as well as smear positive PTB cases chest pain was the leading complaint 67 (40.9 %) followed by night sweat 30 (18.3) and shortness of breath 25 (15.2 %). Then, fever 17 (10.4 %), loss of appetite 17 (10.4 %) and weight loss 8 (4.88) account the rest (Fig. 3).

In the study, 80 (48.8 %) of the study participants had 3 weeks and less duration of cough and 84 (51.2 %) of them had more than 4 weeks. The mean duration of cough was 42 ± 7 days prior to visiting and receiving a treatment. Eighty two (50 %) prisoners visited health institutions and received treatment for TB symptoms. Fourty five (54.9 %) of them visited and got a treatment from prison clinic, and 30 (36.6 %) of them visited civilian clinics and the rest seven (8.5 %) visited both. Moreover, 68 (82.9 %) and 14 (17.1 %) of them had 1–3 times and >3 times visit to clinics because of TB symptoms, respectively. The mean number of visits was 2.5 ± 0.5. On the other hand, half (50 %) of the prisoners did not visit and receive a treatment, even if they had TB symptoms. Their main reasons not to visit the health institutions and get treatment were negligence and inability to get the service.

Sixty two (37.8 %) prisoners were admitted to the prison with TB symptoms; out of which, 25 (40.3 %) of them did not visit and receive a treatment during a pre- and post-imprisonment period. As to their history of contact with a TB patient before imprisonment, 23 (14 %) of them replied for having contact, and 141 (86 %) did not have contact. As to the BMI level, 49 (29.9 %) of them were below 18.5 kg/m^2^, and 115 (70.1 %) of them were above or equal to 18.5 kg/m^2^ (Table 3).

**Table 3:** Univariate analysis of the association between smear positive PTB and morbidity factors among the inmates in Hadiya Zone prison from May to June 2013

Variable	Label	No. tested (%)	No. positive (%)	No. negative(%)	Crude OR (95 % CI)	P value
Duration of cough	≤3 weeks	80 (48.8)	2 (2.5)	78 (97.5)	2.128 (0.523–8.66)	0.532
	>3 weeks	84 (51.2)	1 (1.2)	83 (98.8)	1.0	
Visit to health institution	No	82 (50)	2 (2.4)	80 (97.6)	2.024 (0.498–8.235)	0.568
	Yes	82 (50)	1 (1.2)	81(98.8)	1.0	
Having TB symptoms before imprisonment	No	102 (62.2)	3 (2.9)	99 (97.1)	–	0.173
	Yes	62 (37.8)	0	62 (100)	–	
Body mass index (kg/m^2^)	≤18.5	49 (29.9)	1 (2)	48 (98)	1.0	0.895
	>18.5	115 (70.1)	2 (1.7)	113 (98.3)	0.85 (0.138–7.246)	

In the present study, even though the prevalence of PTB was higher in prisoners who had chest pain, never visited to any clinics for their TB symptoms, visited and got treatment from the prison clinic for less than three times, had no TB symptoms before imprisonment, had never been diagnosed for TB and had no contact with TB patients before their imprisonment, the association was not significant (Table 3).

## Discussion

Prevalence rates of TB in prisons usually exceed the rates in the general population substantially and in average it can reach up to 55 times higher than national prevalence [5]. In agreement to this, in the current study, the point prevalence of smear positive TB in Hadiya Zone Prison was 349.2 per 100,000 populations which was 3.3 times higher than the TB in the general population [3]. However, the prevalence in Hossana was lower than the report from Eastern Ethiopian prisons and North Gondar Zone Prison which was 1913/100,000 and 1482.3/100,000 respectively [8, 9]. The difference could be attributed to the difference in sample size and/or population size and study design as the Eastern Ethiopian study conducted in three prisons which contain 2300 including those TB patients who are on TB treatment already and culture was employed to detect *M. tuberculosis* apart from microscopy. On the other hand, study in Gondar was conducted in a prison containing 1754 prisoners using a light emitting diode (LED) fluorescence microscopy while the present study was conducted in a prison comprising only 859 inmates using compound light microscopy.

Studies from Zambia, Botswana, Russia, Georgia and Thailand showed much higher prevalences [6, 11, 16, 17] On the other hand, lower prevalences were reported from prisons of some Asian and European countries, 259/100,000 in Taiwan, 341/100,000 in Turkey and 215/100,000 in France [18–20]. The relatively lower prevalence in these countries could be due to a good TB control strategy and low TB incidence in the general population as well as in the prisons.

An interesting finding of this study is that smear positive PTB in prisoners from rural areas particularly in farmers was higher than those from urban areas. One possible explanation could be their frequent contact with cattle and more consumption of unpasteurized milk [21]. This is in contrast to the finding of the study in Eastern Ethiopia which showed prisoners from urban areas have higher risk of acquiring PTB than those from rural areas [8]. The study linked the higher risk with relatively high rate of associated HIV infection in urban areas as HIV is the leading modulator of TB infection [22]. Nevertheless, other studies in Tajikistan and different parts of Ethiopia indicated the higher proportion of pulmonary tuberculosis cases in rural prisoners without statistically significant association [23–25].

The current study also identified that all the PTB cases were males. This might be due to small sample size of the female prisoners which may preclude the actual effect of sex, thus making risk comparison inaccurate. However, the male prisoners may be at greater risk of acquiring the infection and become source of transmission, as there is a high overcrowding and poor housing condition compared to their female counterparts. This argument is corroborated by studies carried out among Zambian [6], Malawian [26] and US [27] prisons that documented higher TB prevalence among males than females. Congruent to this observation there were also studies that report male as being a risk factor for TB among Thai [28] and Spanish [29] prisons.

Young adults, who were in the age range between 15–35 years, were found to have higher PTB cases. Although it did not show any level of significant association, a high rate of HIV infection could be one of the possible explanations to the high TB prevalence in this age group. This association was consistently reported among the prison and general population in Africa [30–32]. Likewise, prison studies from high and low TB burden countries documented a high TB prevalence among young adults [6, 26–28].

Prisoners who had no visit from family are significantly at higher risk of acquiring PTB. This could be related to psychosocial support they lack as during stress the adaptive immune system is suppressed due to continually high levels of stress hormones. As a result, the body is less able to produce antibodies and more susceptible to infections [33].

In this study, the length of stay in the prison was not significantly associated with PTB, despite the majority of study participants stayed for the short duration. It was similar to that of Eastern Ethiopian prison and Zambian prison study [6, 8], whereas, Ivory Coast [12] and Cameroon [2] studies indicated a short staying as the risk factor for TB. On the contrary, Spain [29] and Georgian [11] studies reported a longer staying as the risk factor. Although it did not show any level of association in current study, frequent imprisonment could put an individual to repeated exposure of TB infection. This has also been documented usually among individuals who commit crime repeatedly, such as homeless and street gangs that are likely to be deprived of living conditions and health care, thus have greater risk of acquiring TB [27]. Similarly, studies carried out in Spain [29] and Cameroon [7] identified re-imprisonment as the risk factor for TB.

Factors relating to living and crowding conditions did not show any level of significance and hence were not considered as explanatory variables for PTB prevalence in this study, though they are known to favour dissemination of TB. This finding was similar with that of a Zambian study [6]; where there is no differences related to living conditions such as overcrowding, poor dietary conditions and large number of prisoners per cell. In contrary, a case-control study in Russia [10] mentioned prison factors like high ratio of prisoners per available bed, not having own bed clothes, and little time out-doors as independent risk factors. Cross-sectional nature of the study could be one of the possible reasons for not observing the significance level of these factors.

The current study showed that all of the TB positive inmates developed TB symptoms after they joined the prison. Even though there wasn’t any significant association between the symptoms and TB positivity in this study, there are a number of studies that showed significant associations. A Brazilian prison study reported a range of symptoms that had significant association with TB [34]. Similarly, a Georgian study mentioned loss of appetite as an independent risk factor [11]. In Thailand prisons study, weight loss made significant independent contribution to a diagnosis of smear-positive TB [28]. This in conjunction with long cough duration shows an extended lag time before patients get diagnosed and treated rendering the smear positive prisoners to transmit the infection to many others. This could be intensified by the nature of the cells shared by the inmates.

The cells in the study area were poorly ventilated having a single window some of them even not opened throughout the day and accommodate more than one hundred prisoners (the mean number of inmates per cell was 160) who mix all day long with detainees from other cells in enclosed spaces. The lengthy stay of the inmates in the prison could have been rendering the prison to serve as a reservoir of TB transmission. The time left to stay in the prison for most of the TB positive inmates was more than a year which could further enhance transmission of TB.).

The limitations of the study are that it didn’t include prisoners who had a difficulty of getting productive sputum and adequate amount. Similarly, over and under reporting about the risks of PTB by the study participants is highly anticipated in this study. This may have influenced on the value of parameter estimators, such as odds ratio, *P* value and confidence interval; it may have also underestimated or overestimated the prediction of risk factors for PTB. Furthermore, the small sample size of PTB patients could have similar influence on the analysis.

## Conclusion

The current study documented high prevalence of PTB among the prison population; about three times greater than the prevalence in the general population [3]. It also demonstrates a high burden of undetected and infectious PTB cases in the prison. This in conjunction with the favourable nature of prison environment may put the prison population at increased risk of developing the disease. This could also be a great health threat to the surrounding community as TB from inmates may spread through various means. Therefore, the findings of this study should be regarded as a baseline for planning and implementing the prison TB control program, and further studies.

## Abbreviations

AFB: acid-fast bacilli
AIDS: acquired immunodeficiency syndrome
CXR: chest X-ray
DOT: directly observed therapy
HIDN: health, infectious disease and nutrition
HIV: human immunodeficiency virus
LTBI: latent tuberculosis infection
MDR-TB: multidrug-resistant tuberculosis
NTCP: National Tuberculosis Control Program
SNNPR: Southern Nations Nationalities and Peoples’ Region
SSA: Sub-Saharan Africa
TB: tuberculosis
USA: United States of America
WHO: World Health Organization
XDR-TB: extensively drug-resistant tuberculosis
